# Reconfigurable nonlinear photonic activation function for photonic neural network based on non-volatile opto-resistive RAM switch

**DOI:** 10.1038/s41377-022-00976-5

**Published:** 2022-10-06

**Authors:** Zefeng Xu, Baoshan Tang, Xiangyu Zhang, Jin Feng Leong, Jieming Pan, Sonu Hooda, Evgeny Zamburg, Aaron Voon-Yew Thean

**Affiliations:** 1 Integrative Sciences and Engineering Programme, NUS Graduate School, National University of Singapore, Singapore, Singapore; 2 Department of Electrical and Computer Engineering, National University of Singapore, 4 Engineering Drive 3, Singapore, 117583 Singapore

**Keywords:** Integrated optics, Optoelectronic devices and components, Optical techniques, Photonic devices

## Abstract

Photonic neural network has been sought as an alternative solution to surpass the efficiency and speed bottlenecks of electronic neural network. Despite that the integrated Mach–Zehnder Interferometer (MZI) mesh can perform vector-matrix multiplication in photonic neural network, a programmable in-situ nonlinear activation function has not been proposed to date, suppressing further advancement of photonic neural network. Here, we demonstrate an efficient in-situ nonlinear accelerator comprising a unique solution-processed two-dimensional (2D) MoS_2_ Opto-Resistive RAM Switch (ORS), which exhibits tunable nonlinear resistance switching that allow us to introduce nonlinearity to the photonic neuron which overcomes the linear voltage-power relationship of typical photonic components. Our reconfigurable scheme enables implementation of a wide variety of nonlinear responses. Furthermore, we confirm its feasibility and capability for MNIST handwritten digit recognition, achieving a high accuracy of 91.6%. Our accelerator constitutes a major step towards the realization of in-situ photonic neural network and pave the way for the integration of photonic integrated circuits (PIC).

## Abstract (web summary)

A novel nonlinear accelerator based on non-volatile opto-resistive RAM switch was proposed and demonstrated to enable fully in-situ photonic neural network.

## Graphical abstract

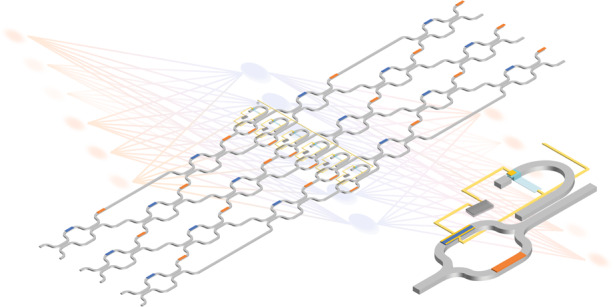

## Introduction

Artificial Neural Network (ANN) is a computational model for mimicking the human brain in information processing^1^. It consists of massive nodes, namely “neurons” connected to each other through synapses. The computational complexity of ANN in model iterations requires large computational ability for multiply-and-accumulate (MAC) operation^2^. With the continuous advancement of ANN, the past decade has witnessed an exponential rise in the demand for high computing speed and low energy consumption^3,4^. As this demand continues, graphics processing unit (GPU) and even central processing unit (CPU)/GPU heterogenous architectures become attractive options for the ANN acceleration since they offer more computational parallelism than CPU^5^. Besides, more electronics architectures have been also developed, such as Application-Specific Integrated Circuit (ASIC) and Field-Programmable Gate Array (FPGA) chips to increase the ANN computing speed and efficiency^6–8^. However, these architectures are still limited by electrical interconnects with resistance and capacitance (RC) parasitic effects and the twilight of Moore’s law for CMOS technology^9^. As an alternative to electronics, photonics has been considered as a promising archetypal solution to address these issues, with ultra-low computation loss, sub-nanosecond latencies and abundant computing parallelism^10,11^. Moreover, photonics can deliver higher bandwidth, better energy-efficiency, and more complex functionality^12^.

Recent works have demonstrated the potential of photonic neural network in the acceleration of ANN. The first photonic ANN was implemented on a free-space light platform with optical lens^13^. However, it has a disadvantage of low integration. Along with the rapid development of integrated photonics, the combination of Micro-Ring-Resonator (MRR)-based weighting bank and Photodetector arrays achieves small-scale matrix multiplication with the assistance of Wavelength Division Multiplexing technology^14,15^, but this method is not efficient enough due to the large footprint of MRRs. To enlarge the matrix computation scale, MZI mesh on an integrated photonic chip has been proposed for MAC operations^16,17^. This corresponds to one of the basic functions of ANN, weighting layer, to interpret incoming signals, with superior propagation speed and power efficiency^18^. However, the lack of another necessary basic function, applying in-situ nonlinear activation function to the sum of weighted inputs after MAC functions, remains an open challenge in photonic neural network. It results in insufficient performance, including low recognition accuracy and slow convergence rate^19^. This originates from the limited and invariable network complexity. Although the number of linear layers can be increased, the linear photonic ANN model still cannot fit the real physical world problems, which hardly follow straightforward linearity.

To address this challenge, several approaches for in-situ nonlinear activation accelerator in photonics have been proposed and extensively investigated, providing suitable paths for achieving a complete suite of ANN in photonics. For example, two-section distributed-feedback (DFB) lasers^20^, vertical-cavity surface-emitting laser (VCSEL)^21^ and disk lasers^22^ have shown promising results, but they are bottlenecked by network scale, frequency of access and power consumption. Moreover, their nonlinear activation responses tend to be fixed during accelerator fabrication, but the nonlinear activation forms should be reprogrammed according to different ANN models and data sets^23^. Thus, as a complementary approach, a more straightforward and flexible implementation is attained by calculating the nonlinear functions in CPU, which connects physical photonic neural networks through electrical-to-optical (E/O) and optical-to-electrical (O/E) converters^24,25^. Unfortunately, it still suffers from the limitations of low efficiency and high latency with frequent access, due to poor performance of parallel computation^26^. Another challenge associated with this approach is the adoption of highly efficient optical-to-electrical and electrical-to-optical converter devices, which greatly influence the power consumption of the whole system^27,28^. Therefore, to address these issues, one should concurrently research both sides: suitable devices to achieve direct communication between photon and electron, as well as efficient and programmable nonlinear activation accelerator structure.

Herein, we have proposed an optical-to-optical nonlinear activation accelerator in an optical-electrical hybrid structure which alleviates the aforementioned challenges on both device and accelerator structure sides. This accelerator has been developed based on a unique Opto-Resistive RAM Switch, whose memristive behaviour is sensitive to incident light, using solution-processed 2D MoS_2_. The solution processed technology has an advantage of the ease of large-scale integration with a low thermal budget, which is critical in processing with highly sensitive optical components on a chip. Furthermore, the Opto-Resistive RAM Switch switching voltage from high resistance state to low resistance state shows a linear dependence to the input optical power, bridging the Opto-Resistive RAM Switch to the photonic ANN for nonlinear activation accelerator. Based on this unique photosensitive device, our proposed accelerator features a variety of nonlinear activation response. The nonlinear accelerator consists of Opto-Resistive RAM Switch, low-power control unit, and MZI with tunable phase change material (PCM). Additionally, this structure allows for the possibility of active tunability of nonlinear response under different initial conditions. In this way, we demonstrate the availability of our Opto-Resistive RAM Switch-based nonlinear activation accelerator in a multi-class MNIST handwritten digit recognition using photonic neural network, with high accuracy and fast convergence rate.

## Results

### Architecture of the novel photonic neural network

**Fig. 1:**
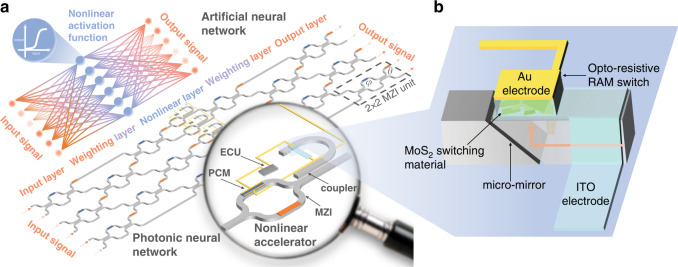
Photonic neural network architecture. **a** The schematic structure of photonic neural network integrated with nonlinear accelerator, which achieves nonlinear activation functions. **b** The schematic of the Opto-Resistive RAM Switch. (ECU Electrical Control Unit, PCM Phase Change Material, MZI Mach–Zehnder Interferometer)

Our overall approach is summarized in Fig. 1. ANN necessitates multiple hidden layers, each with a weighting layer to compute weighting matrix and summation, and a nonlinear layer to execute nonlinear activation function. In the photonic neural network, a programmable MZI mesh contains inner phase-shifters (marked with blue colour) and outer phase-shifters (marked with orange colour) to multiply optical signal from input layer by an assigned weight value and sum over it. Following MZI mesh, nonlinear accelerators apply nonlinear activation functions to the output of the MZI mesh. By repeating such combination of MZI mesh and nonlinear accelerators, photonic neural network achieves in-situ ANN computation with a large number of nodes and connections. The diagram shown in Supplementary Fig. S1 visualises the performance of the photonic neural network equipped with nonlinear accelerators (“PIC + nonlinear accelerator”) against other acceleration architectures for the performance benchmark on ANN acceleration^29,30^. It can be intuitively and conveniently identified that photonic neural network equipped with nonlinear accelerators has better overall performance than other computation architectures, including CPU, GPU, FPGA, ASICs and PIC.

MZI-mesh based weighting layer is configured with some 2 × 2 MZIs as marked with a dash box in Fig. 1a. It has been demonstrated that MZI unit can perform all rotations in unitary group of degree two, U(2), by adjusting PCMs *θ* and *φ*^31,32^. In this regard, any weighting matrix can be decomposed into the product of several U(2)s. Thus, MZI mesh is capable of adding any weighting matrix into optical input. The unitary transformation U(2) of MZI can be given by^33^

1 $$U_{MZI} = \frac{1}{2}\left[ {\begin{array}{*{20}{c}} {e^{i\theta }\left( {e^{i\varphi } - 1} \right)} & {e^{i\theta }\left( {e^{i\varphi } + 1} \right)} \\ {i\left( {e^{i\varphi } + 1} \right)} & {1 - e^{i\varphi }} \end{array}} \right] = \left[ {\begin{array}{*{20}{c}} {u_{11}} & {u_{12}} \\ {u_{21}} & {u_{22}} \end{array}} \right]$$

where *θ* and *φ* are the phase shifts in PCMs (Fig. 1a). The detail of proposed nonlinear accelerator is shown in the magnified view of the nonlinear layer. It contains an optical coupler to split a fraction of light into the bent sub-waveguide from the main waveguide route, a micro-mirror to divert light into the top of sub-waveguide, a Opto-Resistive RAM Switch with MoS_2_ switching material to capture the optical information in terms of optical power and incident wavelength, an electrical control unit (ECU) to drive Opto-Resistive RAM Switch and MZI simultaneously, and a MZI with PCM to achieve a feedback loop modulating the light passing through the main route. The principle of its operation will be explained later in the article. There is no need for extra footprint space for control unit compared with other methods introduced above, since control unit is small enough that can just occupy gaps within photonic network. Here, Opto-Resistive RAM Switch, integrated with micro-mirror, plays a key role in the accelerator function. The schematic of Opto-Resistive RAM Switch is shown in a detailed view in Fig. 1b. Opto-Resistive RAM Switch consists of an ITO-MoS_2_-Au sandwich-like structure (Supplementary Fig. S2).

### Opto-Resistive RAM Switch characteristic

Opto-Resistive RAM Switch employs solution-processed MoS_2_ switching material, which is a film spin-coated on the bottom electrode from a MoS_2_ high-concentrated ink. The ink is prepared through ion-intercalation-driven exfoliation of a MoS_2_ bulk. However, MoS_2_ should meet requirements on thickness (1–5 nm) and roughness (≤2 nm) to avoid excessive driving voltage and optical loss and should enable incident-angle-independent absorption at certain wavelength. Surface morphology of stack of 2D MoS_2_ sheets measured using Atomic Force Microscopy (AFM) is shown in Supplementary Fig. S3. AFM-image demonstrates that MoS_2_ film has low roughness of 1.2 nm, which meets the low refraction loss requirement of fabricating Opto-Resistive RAM Switch^34^. This MoS_2_ synthesis technology allows Opto-Resistive RAM Switch to be fabricated on the top of sub-waveguide. The Raman spectra, collected from MoS_2_ film on SiO_2_/Si substrate, shows strong peaks at 383.5 cm^−1^ (E^1^_2g_) and 408.2 cm^−1^ (A_1g_) (Supplementary Fig. S4), which are consistent with previous reports^35,36^, and it indicates the multi-layered structure of the MoS_2_ 2D sheets. Moreover, MoS_2_ exhibits incident-angle-independent absorption of light at wavelengths <600 nm (Supplementary Figs. S5–6). The analyses above raise a possibility of integrating Opto-Resistive RAM Switch with integrated photonic circuit. For accurate resistance switching characterization, an Opto-Resistive RAM Switch device is prepared on SiO_2_/Si substrate.

**Fig. 2:**
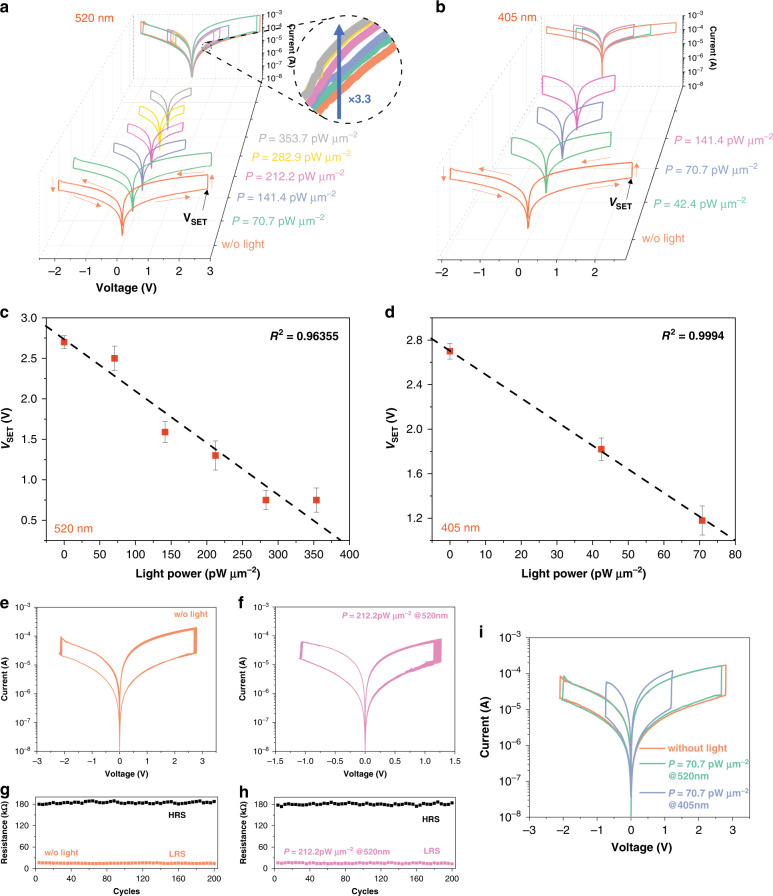
Electrical characteristic of Opto-Resistive RAM Switch. **a**, **b** Current-voltage photoresponse characteristic of Opto-Resistive RAM Switch under a series of illumination from 0 to 353.7 pW·μm^−2^ at the wavelength of (**a**) 520 nm and (**b**) 405 nm, respectively. The inset shows the zoom-in of the *I*–*V* curves before resistance switching happens. **c**, **d** Mean V_SET_ variation as a function of applied optical power at (**c**) 520 nm and (**d**) 405 nm, respectively. Note that the error bars are the standard error on the mean of 200 cycles. The black dash lines are fitted straight lines. **e** I–V characteristic of Opto-Resistive RAM Switch under 200 cycles without input optical signal. **f** I–V characteristic of Opto-Resistive RAM Switch under 200 cycles with 212.2 pW·μm^−2^ optical power at 520 nm. **g**, **h** The statistics and distribution of high resistance state and low resistance state over 200 cycles (**g**) without light and (**h**) with 212.2 pW·μm^−2^ optical power at 520 nm. **i** The I–V photoresponse characteristic of Opto-Resistive RAM Switch under 70.7 pW·μm^−2^ illumination with different input wavelengths. (HRS High Resistance State, LRS Low Resistance State)

Figure 2a, b shows the bipolar resistance switching characteristics of Opto-Resistive RAM Switch activated by different optical power of 520 nm and 405 nm guided light, respectively. For the typical current-voltage (I-V) measurement without light input (orange lines in Fig. 2a, b), a DC voltage is applied to the Au top electrode and the ITO bottom electrode is grounded. During the voltage sweep from 0 to 3 V, an obvious abrupt increase of current can be observed while applied voltage reaches a threshold voltage, which is defined as V_SET_ (e.g. V_SET_ ≈ 2.7 V without illumination), and Opto-Resistive RAM Switch is switched from high resistance state to low resistance state. In the reversed sweep, negative voltage (−2.2 V) makes Opto-Resistive RAM Switch completely return to high resistance state, termed as RESET process. The V_SET_ signifies that at this voltage the electrical resistance state of Opto-Resistive RAM Switch, with capacity of non-volatile memory, can be changed as previously reported resistance switching devices^37,38^. This switching characteristic is conducted under different optical power with a fixed wavelength irradiance as shown in Fig. 2a, b. The light is absorbed in the MoS_2_ material after transmission through bottom ITO electrode, as the photon energy of 2.38 eV and 3.06 eV are larger than the bandgap of MoS_2_ material at room temperature (1.29–1.88 eV). Carrier concentration increases with increasing optical power that leads to the increase of high resistance state current with fixed wavelength (inset in Fig. 2a). Remarkably, during the SET process, V_SET_ steadily decreases from 2.7 to 0.6 V with the increased optical power from 70.7 to 282.9 pW·μm^−2^ at 520 nm wavelength, followed by a saturation of V_SET_. The similar phenomenon can be observed for 405 nm wavelength illumination as shown in Fig. 2b: V_SET_ declines from 2.7 to 1.2 V with increased optical power from 0 to 70.7 pW·μm^−2^ before a saturation of V_SET_. This effect related to input optical power is summarized in Fig. 2c, d for 520 nm and 405 nm, respectively, and it can be fitted perfectly in straight line with high coefficient of determination (R^2^), 0.9635 and 0.9994 for 520 nm and 405 nm respectively. This linear relationship can be expressed as,

2 $$V = kP_{abs} + b$$

where *k* is the slope, *P*_*abs*_ is absorbed optical power of Opto-Resistive RAM Switch, and *b* is the intercept. This allows the optical power to be converted into the electrical signal (V_SET_) linearly. As for the working function in the process of the acceleration, the response of Opto-Resistive RAM Switch is nonlinear since briefly it is a sudden change of output in terms of current, which is a necessary signal driving the accelerator. Thus, Opto-Resistive RAM Switch’s optical characteristic is unique and different from normal photodetectors^39,40^, which detect and convert the optical power into current in a linear way. The unique characteristic of our Opto-Resistive RAM Switch is critical to to the realization of the nonlinear activation accelerator.

As discussed above, the frequent access to nonlinear activation accelerator requires that Opto-Resistive RAM Switch can maintain its switching characteristic in many cycles. Furthermore, the resolution (R) of Opto-Resistive RAM Switch depends on the variation of its characteristic at each optical power input, which is defined as bellow,

3 $$\begin{array}{l}R = \mathop {{{{{\mathrm{argmax}}}}}}\limits_n \left| {\left\{ {V_1,V_2,V_3,V_4, \ldots V_n \in V_r} \right\}} \right|,V_i \cap V_j\\\quad = \emptyset ,{{{\mathrm{i}}}}\, \ne \,{{{\mathrm{j}}}} \,\le\, {{{\mathrm{n}}}}\end{array}$$

where |*x*| represents the number of elements in a set *x. V*_*i*_ means the V_SET_ variation of the *i*^th^ input power state, and *V*_*r*_ corresponds to the range of possible V_SET_. To maximize the power perception resolution, the variation of V_SET_ at each optical power input should be as small as possible. Cycle-to-cycle evaluation of the Opto-Resistive RAM Switch at room temperature has been carried out. As shown in Fig. 2e–h, the Opto-Resistive RAM Switch exhibits stable and uniform switching over 200 cycles with negligible cycle-to-cycle variation in resistance states and switching voltages under both dark (Fig. 2g) and light circumstances (Fig. 2h) Moreover, the variation of V_SET_ ranges from 0.03 to 0.08 V for different optical input power, which means Opto-Resistive RAM Switch can differentiate up to 39 optical power independent states. Fig. 2i shows the comparison of switching characteristic for different input wavelength but with the same optical power at 70.7 pW·μm^−2^. Obviously, higher input photon energy induces lower V_SET_ and smaller switching window.

### Opto-Resistive RAM Switch operation mechanism

**Fig. 3:**
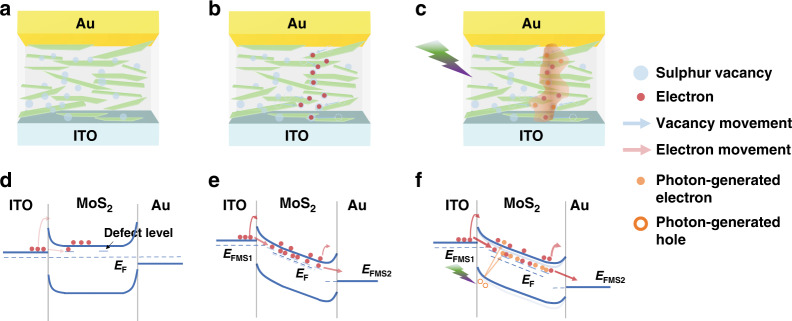
Mechanism of Opto-Resistive RAM Switch. **a**–**c** The conducing states in the Opto-Resistive RAM Switch at (**a**) initial state, **b** SET process without optical input, and (**c**) SET process with optical input. **d**–**f** Corresponding energy band diagram of Opto-Resistive RAM Switch at (**d**) initial state, **e** SET process without optical input, and (**f**) SET process with optical input

The resistance switching characteristic and optical response are contributed to the vacancy migration and photon-induced heat generation. The resistance switching processes are explained in Fig. 3a–c and corresponding energy band diagrams at different states are shown in Fig. 3d–f. For the MoS_2_ solution-processed material, sulphur vacancies are created at the edge of each 2D sheets during solution-exfoliation process as evidenced by our previous work^41^. The electron affinity of MoS_2_ is around 3.0 eV^42^, lower than work functions of Au and ITO (5.1 eV and 4.7 eV, respectively)^43^, leading to the formation of Schottky barrier contacts on both interfaces of Au/MoS_2_ and MoS_2_/ITO. In this case, only few electrons can pass over or tunnel through the barrier and no sulphur vacancies filament is formed. In the SET process, the external bias reduces the width and height of Schottky barrier and therefore increases the electron thermal emission and tunnelling probability, resulting in the improved current. Simultaneously, the positively charged sulphur vacancies migrate along the edge of MoS_2_ sheets under voltage bias, bridge the top and bottom electrodes and finally form a conducive path across the MoS_2_ layers. The resistance states transit from the high resistance state to low resistance state due to much increased tunnelling electrons with higher vacancy defect concentration (quasi-continuous defect level) in the pathway. For photon-response behaviour of Opto-Resistive RAM Switch, by absorbing photons in the interfaces, photoelectric effect creates electron-hole pairs, and the generated electrons are excited into sulphur vacancies defect level and conductance band in the room temperature. Besides, photogating effect that originates from trapped photogenerated electrons can further lower the Schottky barriers^44^. Thus, under illumination, the current increases with increasing carrier concentration (3.3 times as shown in the inset of Fig. 2a) and it produces more heat from joule heating. Current-induced Joule heating and optical power dissipation accelerate the sulphur vacancies movement to form the defect level with higher concentration. It reduces the dependency on external bias and thus V_SET_ decreases under illumination.

### Accelerator structure based on Opto-Resistive RAM Switch

**Fig. 4:**
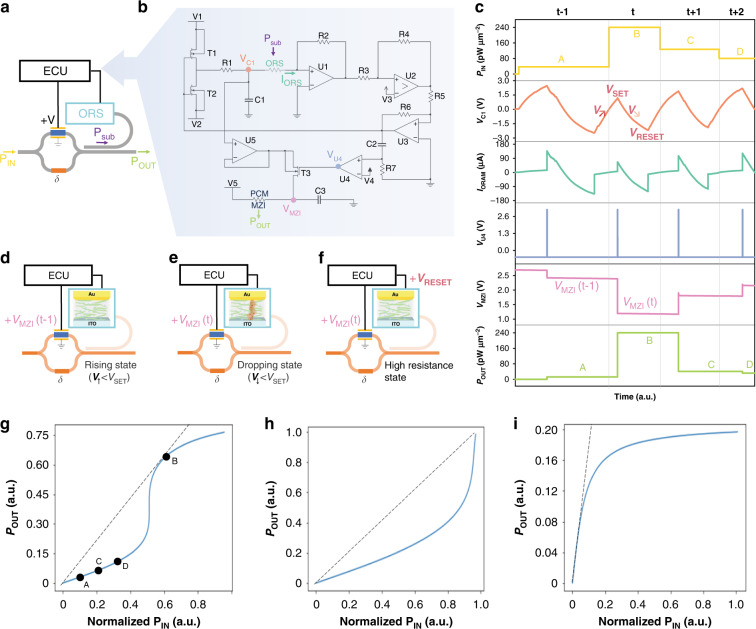
Accelerator structure and function. **a** Simplified schematic of the proposed nonlinear accelerator, corresponding to Fig. 1b. **b** Circuit diagram of electrical control unit with marked nodes. **c** Time-series diagram of data obtained in marked nodes over 4 cycles. **d**–**f** Working states in nonlinear accelerator at (**d**) rising state, **e** dropping state and (**f**) high resistance state. **g**–**i** Nonlinear activation functions output transmission power as a function of normalized input signal power. **g**–**i** correspond to sigmoid, softplus and clamped ReLU responses respectively. (ORS Opto-Resistive RAM Switch, ECU Electrical Control Unit, MZI Mach–Zehnder Interferometer, PCM Phase Change Material)

Due to the ability of photon-sensitive nonlinear switching, Opto-Resistive RAM Switch plays an important role in photon-electron communication in the nonlinear accelerator. Schematically the accelerator structure shown in Fig. 1b can be represented by Fig. 4a, where the grey lines and black lines represent optical waveguides and electrical pathways, respectively. At the beginning, optical signal propagating through MZI (P_sub_) enters a directional coupler which couples a portion (*β*) of signal into Opto-Resistive RAM Switch through bent sub-waveguide. The Opto-Resistive RAM Switch absorbs the light with absorption coefficient (*α*) and switches the resistance at V_SET_, which is an indicator of the P_abs_ with linear relationship. Here, we assume input optical signal is with electric field intensity (E) and the corresponding optical power is given by

4 $$P = \frac{{ab}}{4}E^2\frac{1}{{Z_{TE}}}$$

5 $$Z_{TE} = \frac{{\upeta }}{{\sqrt {1 - \left( {\lambda /\lambda _C} \right)^2} }}$$

6 $${\upeta} = \sqrt {\mu /\varepsilon }$$

7 $$\lambda _C = 2a$$

where *a* and *b* are width and depth of the rectangular waveguide respectively, *ε* is dielectric constant, *μ* is magnetic permeability. The voltage driving Opto-Resistive RAM Switch is provided by electrical control unit, whose circuit constitution is given by Fig. 4b. Positive (V1) and negative (V2) power supplies power the Opto-Resistive RAM Switch through a reversed switch-pair, constituted by a PMOS transistor (T1) and a NMOS transistor (T2), after a specified RC delay (*τ* = R1C1, where τ is RC time constant). Next, it is followed by a trans-impedance amplifier (U1) to convert current into voltage, a hysteresis comparator (U2) to judge the state of Opto-Resistive RAM Switch (low or high resistance state), and a voltage reverser (U3). Initially increasing voltage V_C1_ is applied to Opto-Resistive RAM Switch with T1 on and T2 off, and while the current of Opto-Resistive RAM Switch (I_ORS_) suddenly increased due to the Opto-Resistive RAM Switch switching under illumination, output voltage of U3 reverses and induces T1 off and T2 on. In this case, V_C2_ starts to be pulled down by V2. Besides, simultaneously, another route generates a pulse activated by reversed output of U3 through a specified RC delay (*τ* = R7C2) and a comparator (U4). This pulse opens one transistor switch (T3) within the pulse time to “read” the maximum voltage of V_C1_ (V_SET_) using a voltage follower (U5) and this V_SET_ is applied back to PCM on one arm of MZI to modulate the light go through the main route. The electrical modulation of MZI can be calculated as

8 $$\vec E_o = \frac{{\vec E_I}}{2}\left( {e^{ - j\left( {\frac{{\pi V}}{{V_\pi }}} \right)} + e^{ - j\delta }} \right)$$

9 $$V_\pi = \frac{\lambda }{{n^3}}\frac{1}{r}\frac{d}{L}$$

where $\vec E_I$ and $\vec E_o$ are the input and output electrical fields of MZI respectively and *V*_*π*_ is the half-wave voltage, which causes phase change π of phase shifter. And *λ* is the input wavelength, *n* is the corresponding refractive index, *r* is electro optic coefficient, *L* is the length of interferometric arms and *d* is the thickness of PCM. Combining the expressions above, the mathematical form of nonlinear activation function achieved by nonlinear accelerator can be written explicitly as

10 $$P_O = \frac{{P_I}}{2}cos^2\left( {\frac{{\frac{{\pi \left( {k\alpha \beta P_o + b} \right)}}{{V_\pi }} + \delta }}{2}} \right)$$

To explain the process of such runtime architecture intuitively, time-series diagram is plotted in Fig. 4c and Supplementary Fig. S7. While V_C1_ increases before reaching at V_SET_ (t) (Fig. 4d), V_MZI_ (t-1) is applied constantly to PCM. Until V_SET_ changes the state of Opto-Resistive RAM Switch, V_MZI_ (t-1) suddenly turns into V_MZI_ (t) controlled by one pulse of V_U4_. Subsequently, it is followed by a decreasing V_C1_ (Fig. 4e) to V_RESET_, at which Opto-Resistive RAM Switch switches back from low resistance state to high resistance state but V_MZI_ (t) is still held until next cycle of resistance switching in Opto-Resistive RAM Switch (Fig. 4f). As shown in Fig. 4c, a perfect response of input optical signal in several loops can be viewed, and such nonlinear accelerator easily satisfies one important requirement for photonic neural network: response frequency (voltage sweeping frequency) must be higher than optical signal changing frequency, since the voltage sweeping frequency depends on controllable R1C1 delay. The formula for sweeping voltage is given by

11 $$V_{C1} = \left\{ {\begin{array}{*{20}{c}} {(V_1 - V_2)\left( {1 - e^{\frac{{ - t}}{{R1C1}}}} \right) + V_2,V_{RESET} < V_{C1 \uparrow } < V_{SET}} \\ {(V_2 - V_1)\left( {1 - e^{\frac{{ - t}}{{R1C1}}}} \right) + V_1,V_{RESET} < V_{C1 \downarrow } < V_{SET}} \end{array}} \right.$$

Moreover, a benefit of having an adjustable PCM (*δ*) in another arm of MZI as shown in Fig. 4a is that, in principle, this nonlinear accelerator can be programmed to synthesize different activation functions. Figure 4g–i show various nonlinear activation functions, sigmoid, softplus and clamped rectified linear unit (ReLU), at different initial *δ* values. Notably, every loop in Fig. 4c corresponds to different states of nonlinear function in Fig. 4g. This reconfigurability opens up the possibility of selecting suitable nonlinear functions for different specific tasks and distinguishes this method from previous nonlinear function approaches^20,45^.

## Discussion

**Fig. 5:**
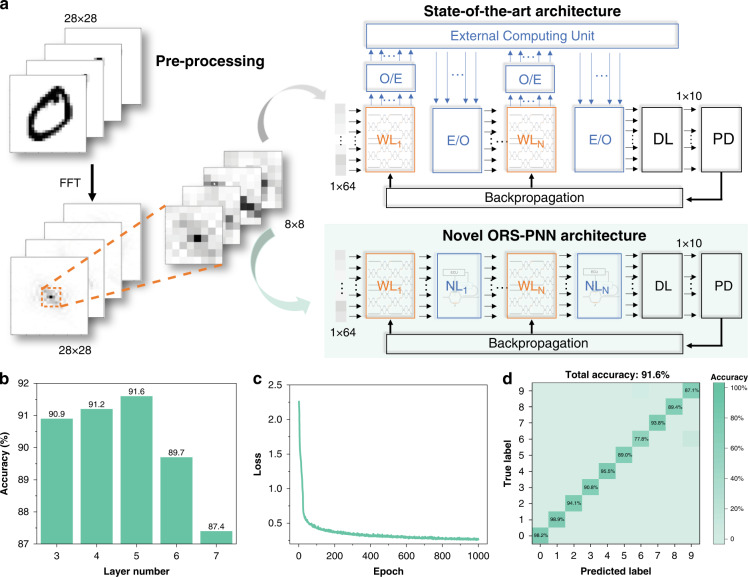
Photonic neural network with nonlinear accelerator. **a** Schematic illustration of MNIST handwritten digit classifier based on photonic neural network accelerated by Opto-Resistive RAM Switch-based nonlinear accelerator. **b** the testing accuracy of photonic neural network architecture on MINST dataset, as a function of layer number. **c** The calculated loss at each epoch during the learning procedure with five layers. **d** The confusion matrix of photonic neural network. (ORS-PNN Photonic Neural Network with Opto-Resistive RAM Switch-Based Nonlinear Accelerator, O/E Optical-to-Electrical Convertors, E/O Electrical-to-Optical Convertors, WL Weighting Layer, NL Nonlinear Layer, DL Drop Layer, PD Photodetectors)

To validate the functionality of the proposed nonlinear accelerator, a fully connected photonic neural network using Opto-Resistive RAM Switch-based nonlinear accelerator is implemented in the simulation. The schematic of this network for the MNIST handwritten digits classification task is shown in Fig. 5a. This MNIST dataset contains 70,000 greyscale images with 28 × 28 pixel, which is a representative database for neural network model training.

To reduce the input data dimension, Fast Fourier Transform (FFT) and edge-removal are used to convert real images into k-space images. The FFT of 2D image is given by the following equation

12 $$F\left( {k_x,k_y} \right) = \mathop {\sum}\limits_{m = 0}^{M - 1} {\mathop {\sum}\limits_{n = 0}^{N - 1} {f(m,n)e^{ - j2\pi \left( {k_x\frac{m}{M} + k_y\frac{n}{N}} \right)}} }$$

where *F*(*k*_*x*_, *k*_*y*_) is the value of the images in frequency domain corresponding to the coordinates *k*_*x*_ and *k*_*y*_, *f*(*m*, *n*) is the real pixel at coordinates (*m*, *n*), and *M* and *N* are the dimensions of the image. The dimension of images is unchanged (28 × 28) after FFT, and the features of images experience centralization since FFT represents spatial frequency distribution of grey level gradients with the lowest frequency in the centre and the highest frequency at four corners. Afterwards, removal of fours edges in each image reduces the dimension from 28 × 28 into 8 × 8 but preserves most of frequency features. The reasons for using FFT include not only dimensionality reduction but also the feasibility of FFT in integrated photonics^46,47^.

At the input of photonic neural network using Opto-Resistive RAM Switch-based accelerator, input images in a form of 8 × 8 pixel array are reconfigured into 64 × 1 array. This photonic neural network starts from several staggered weighting layers (WL) and nonlinear layers (NL) to drop layer (DL), which maps 64 inputs into 10 outputs for ten dights recognition. At the end, photodetectors (PD) convert optical signal into electrical signal for backpropagation calculation, which will optimize weighting layers in the training process. It is worth mentioning, here, the nonlinear layer adopts softplus nonlinear function as shown in Fig. 4h. On account of using nonlinear accelerator, this photonic neural network architecture is more efficient and simplified compared with other photonic neural networks in previous works^16^, which consume more energy and generate more delay during optical-to-electrical and electrical-to-optical conversions. And the previous methods are limited by on-chip space or complexity of network connection with CPU. Specifically, compared with previous methods for nonlinear activation function, our accelerator reduces the average power consumption by 20.2× and shrinks the footprint by around 40%.

To observe the dependence of recognition accuracy on the layer number, Fig. 5b shows the testing accuracy of the photonic neural network with different number of weighting-nonlinear layers. The accuracy reaches a peak at 91.6% with 5 weighting-nonlinear layers. The corresponding loss has an abrupt dropdown, equivalently fast iteration, before 50 epochs with a batch size of 500 in network training as shown in Fig. 5c. The confusion matrix for 5-layer photonic neural network computed over the testing dataset (Fig. 5d) shows the correct prediction for each digit image. Overall, these demonstrate the possibility of accelerating photonic neural network using proposed Opto-Resistive RAM Switch-based nonlinear accelerator.

This nonlinear accelerator based on MoS_2_ Opto-Resistive RAM Switch provides a promising approach for the realization of in-situ photonic neural network. Meanwhile, its simple architecture, low energy consumption and small chip size make it practical to have a wide field of application with good prospects. It can be further extended into the acceleration of more types of neural network that in photonics there has been a number of research works about, such as convolutional neural networks^48^, recurrent neural networks^49^ and long short term memory networks^50^. Moreover, with the incorporation of Wavelength Division Multiplexing technology, it may be capable of computing with high parallelism using different wavelengths, as shown in Fig. 2i.

In conclusion, we have developed a programmable nonlinear accelerator based on Opto-Resistive RAM Switch, which consists of solution-processed MoS_2_. By cleverly leveraging the linear relationship that exists between the input optical power and the voltage that leads to abrupt resistance switching, Opto-Resistive RAM Switch proves the advantage of having the unique functionality to perform as a nonlinear switch that is critical to the functionality of the accelerator, compared to typical photonic components, like photodetector. Using this novel Opto-Resistive RAM Switch, our proposed nonlinear accelerator offers remarkable flexibility to use, because it allows generation of different nonlinear activation functions programmatically. The implementation of our nonlinear accelerator surpasses the limitation of outsourced nonlinear activation functions and achieves a comparable classification accuracy and fast iteration on an in-situ fully connected photonic neural network for MNIST classifier application. On the other hand, from a viewpoint of architecture, our nonlinear accelerator has the potential to significantly outperform the previous nonlinear activation architectures in terms of energy efficiency and complexity. In addition, it is very compact with small footprint. It paves the way for promising in-situ photonic neural network with ultra-high computation speed and parallelism.

## Materials and methods

### Solution-processed MoS_2_ preparation

High-quality semiconducting MoS_2_ nanosheets were fabricated with an electrochemical intercalation assisted exfoliation method^51^. Subsequently, the exfoliated MoS_2_ nanosheets were dispersed in isopropanol to obtain the final MoS_2_ ink, which as used for device fabrication.

### Opto-Resistive RAM Switch fabrication and characterization

Solution-processed MoS_2_ is spin-coated on p-Si wafer with 90 nm SiO_2_ layer, followed by electron beam lithography and rapid thermal annealing. The surface height image is characterized by Atomic Force Microscopy and the Raman spectroscopy. ITO (40 nm) was deposited by sputtering system followed by lithography patterning and ICP-RIE etching to form electrodes. Top Au electrode (40 nm) is formed by electron beam photolithography and deposition using electron beam evaporator followed by lift-off process. The electrical and optical measurements were conducted by Agilent parameter analyzer B1500A and Lakeshore Cryogenic probe station with fixed-wavelength lasers.

### Accelerator and photonic neural network simulation

Accelerator architecture function is analysed using co-simulation of Cadence PSpice design tool and Synopsys OptSim platform. The Neuroptica Python package is used for photonic neural network simulation. In MNIST digit classification task, input port number of MZI mesh is set to 64.

## Supplementary information

Supplementary Information

## Data Availability

The main data supporting the findings of this study are available within the article. Extra data are available from the corresponding authors on reasonable request.
