# Supplementary material for: Reconfigurable nonlinear photonic activation function for photonic neural network based on non-volatile opto-resistive RAM switch
Source: Light Sci Appl. 2022 Oct 6;11:288. doi: 10.1038/s41377-022-00976-5 (PMC9537414; doi:10.1038/s41377-022-00976-5)
Supplement: Supplementary file 1 — Supplementary Information [file 41377_2022_976_MOESM1_ESM.docx]

Supplementary Information for

**Reconfigurable Nonlinear Photonic Activation Function for Photonic Neural Network Based on Non-Volatile Opto-Resistive RAM Switch**

Zefeng Xu^1,2*^, Baoshan Tang^2^, Xiangyu Zhang^2^, Jin Feng Leong^2^, Jieming Pan^2^, Sonu Hooda^2^, Evgeny Zamburg^2^, Aaron Voon-Yew Thean^1,2*^

1. Integrative Sciences and Engineering Programme, NUS Graduate School, National University of Singapore

2. Department of Electrical and Computer Engineering, National University of Singapore, 4 Engineering Drive 3, Singapore 117583, Singapore.

*email: [xuzefeng@u.nus.edu](mailto:xuzefeng@u.nus.edu); [Aaron.Thean@nus.edu.sg](mailto:Aaron.Thean@nus.edu.sg)

**
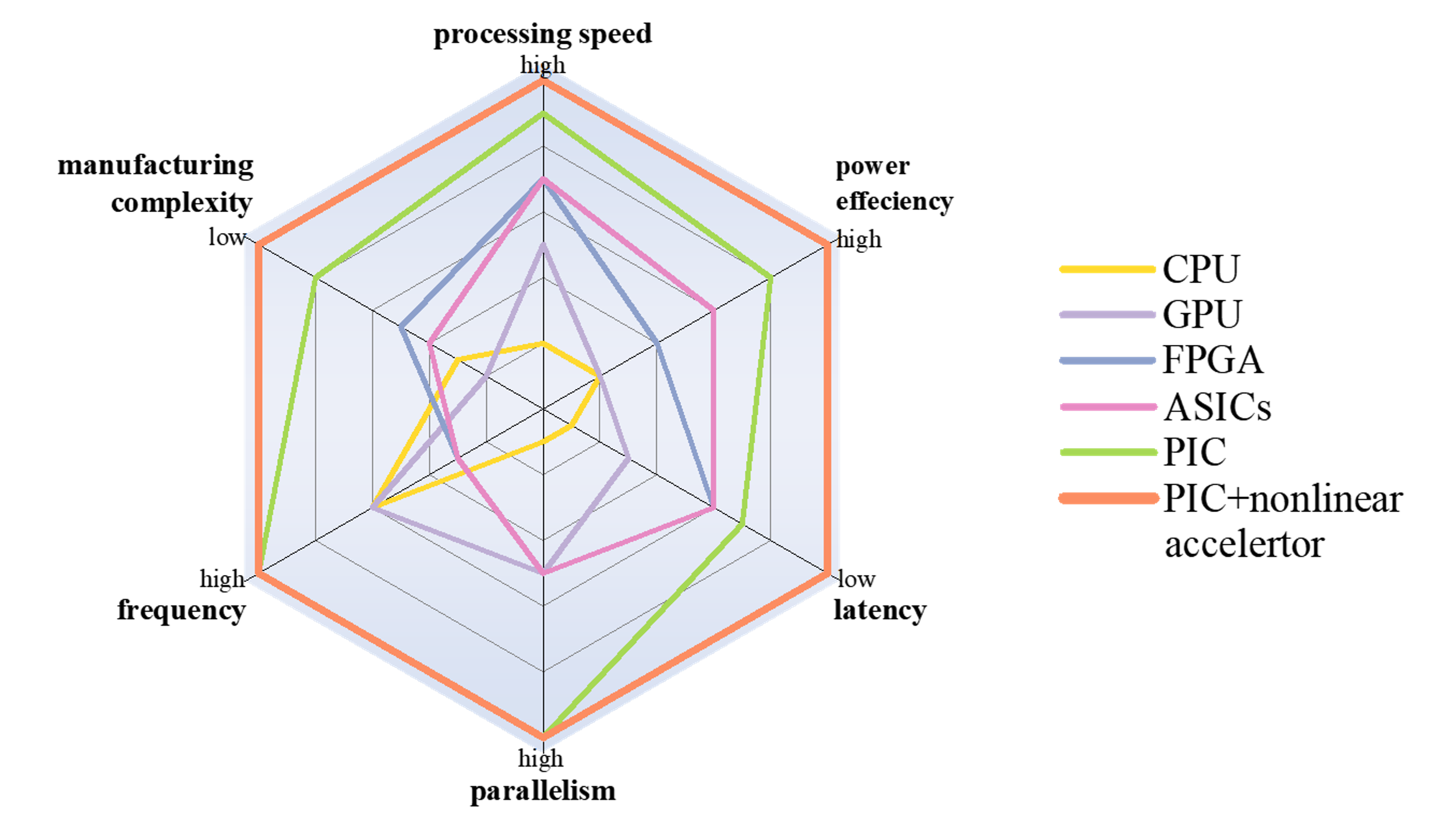
**

**Fig. S1** The performance comparison on benchmark^1, 2^.


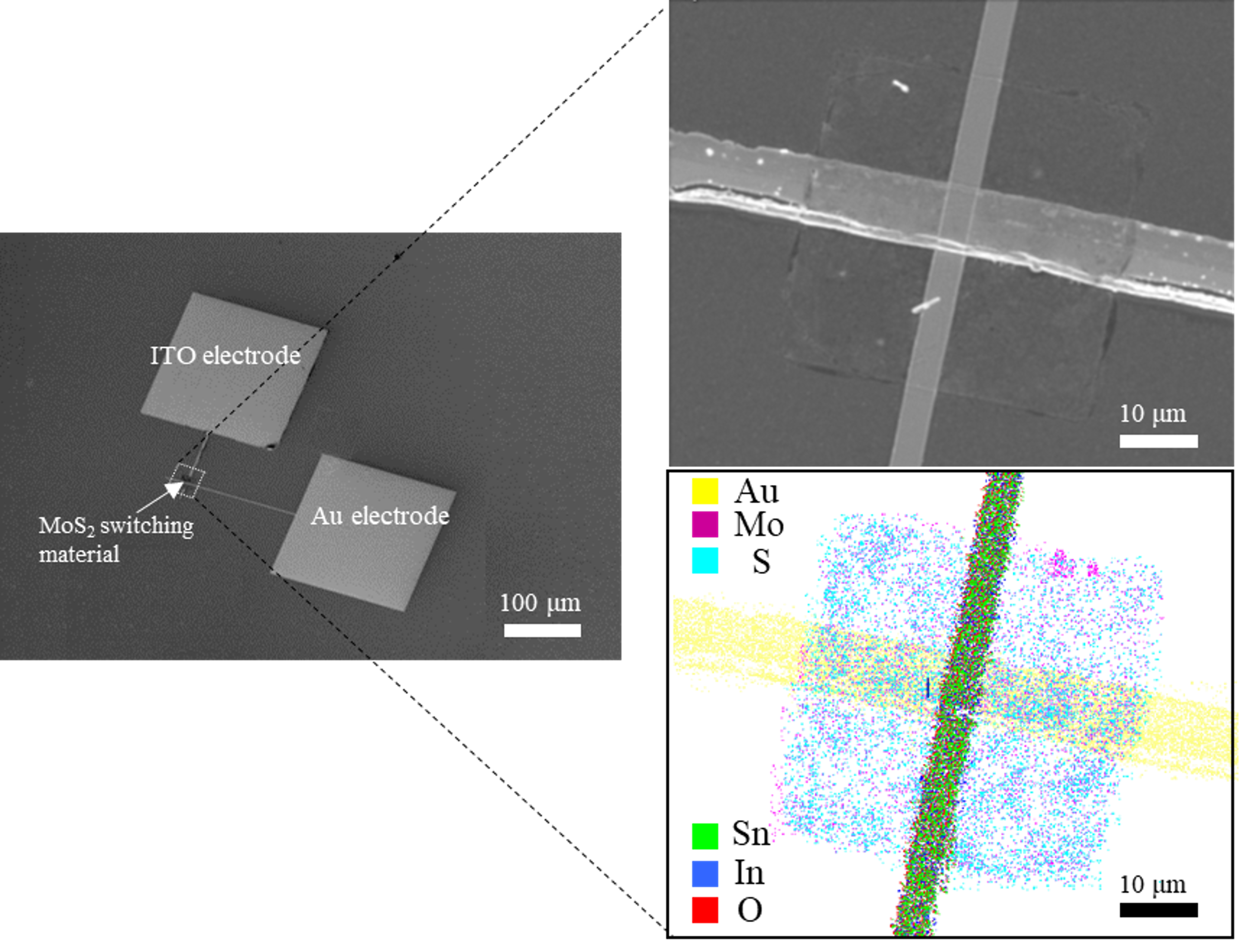


**Fig. S2** Scanning Electron Microscope (SEM) and corresponding Energy-Dispersive X-Ray Spectroscope (EDS) images of an ORS device.


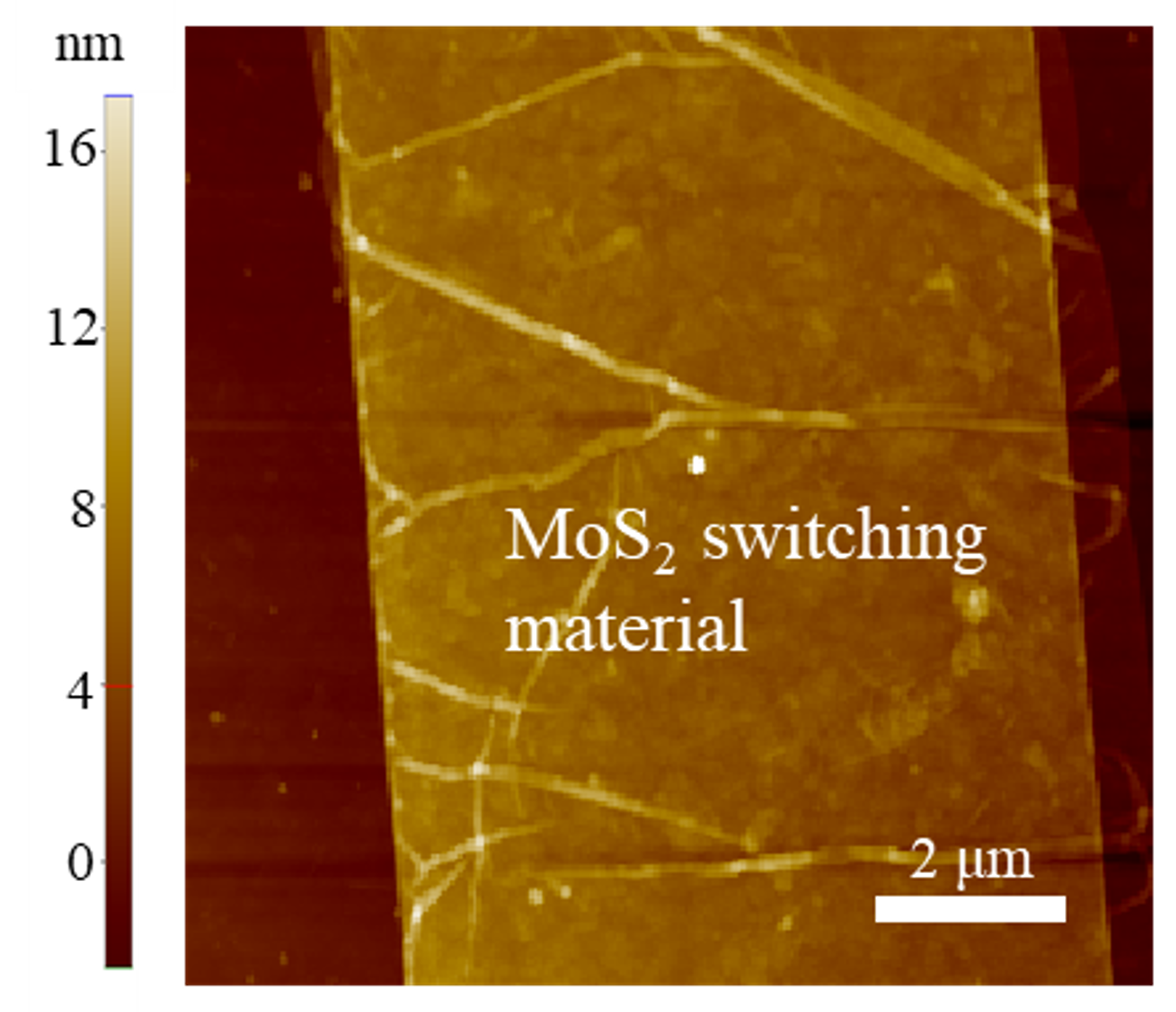


**Fig. S3** AFM image of patterned spin-coated solution-processed MoS_2_ active material.


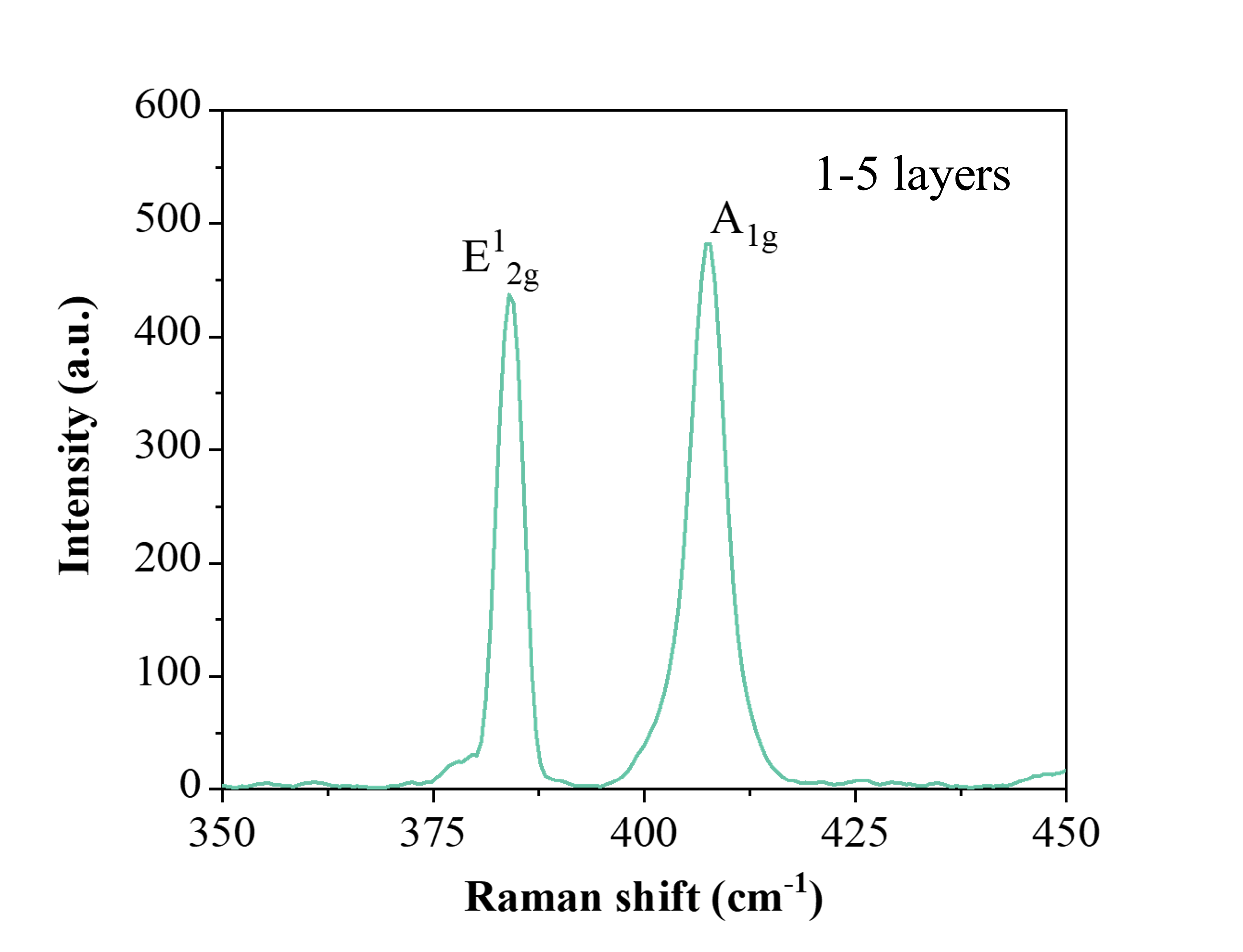


**Fig. S4** Raman spectra of spin-coated solution-processed MoS_2_ active material.

**
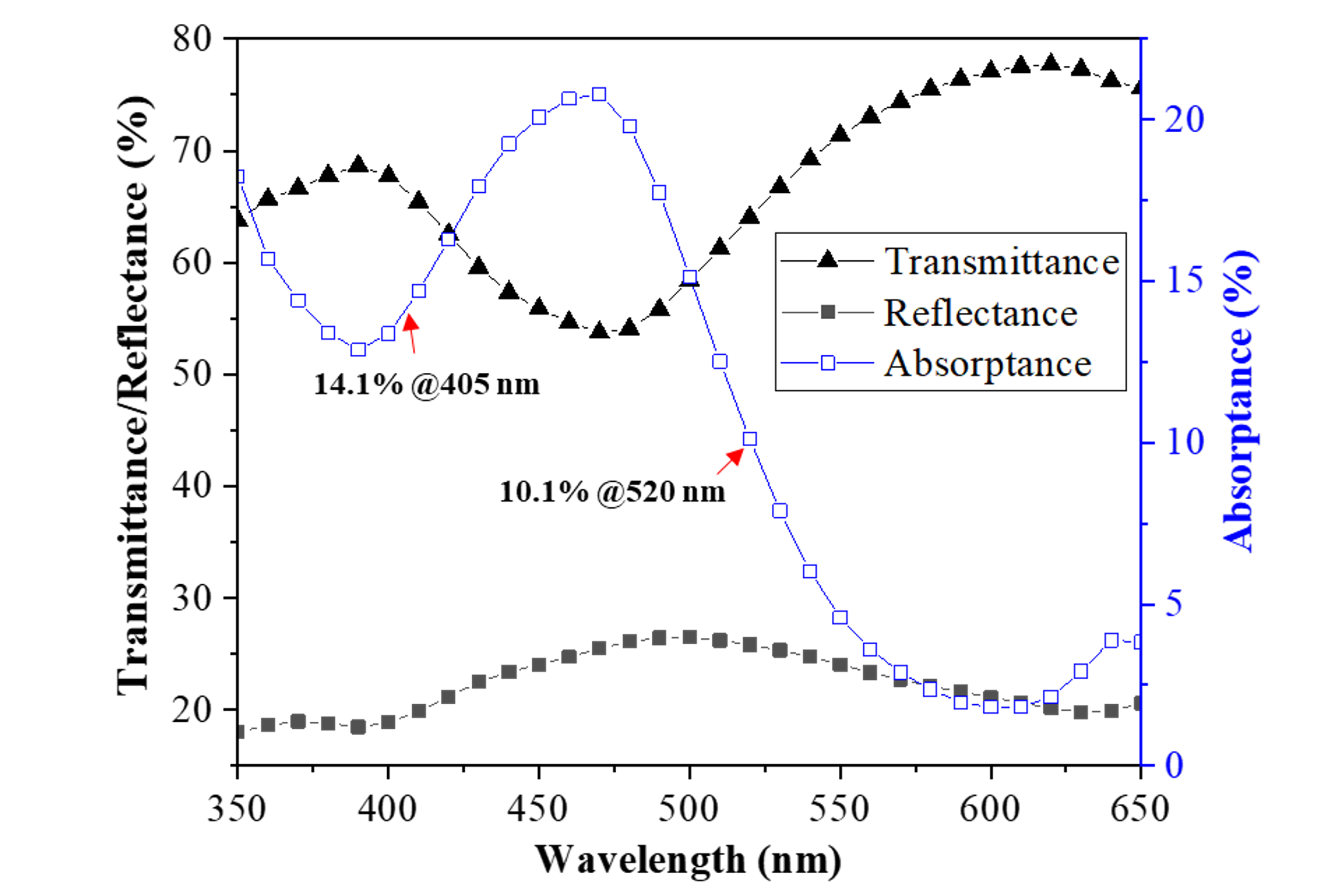
**

**Fig. S5** Transmittance, reflectance and absorptance spectra of spin-coated solution-processed MoS_2_ active material.

**Fig. S6** Absorbance spectra as a function of wavelength with different incident angles.


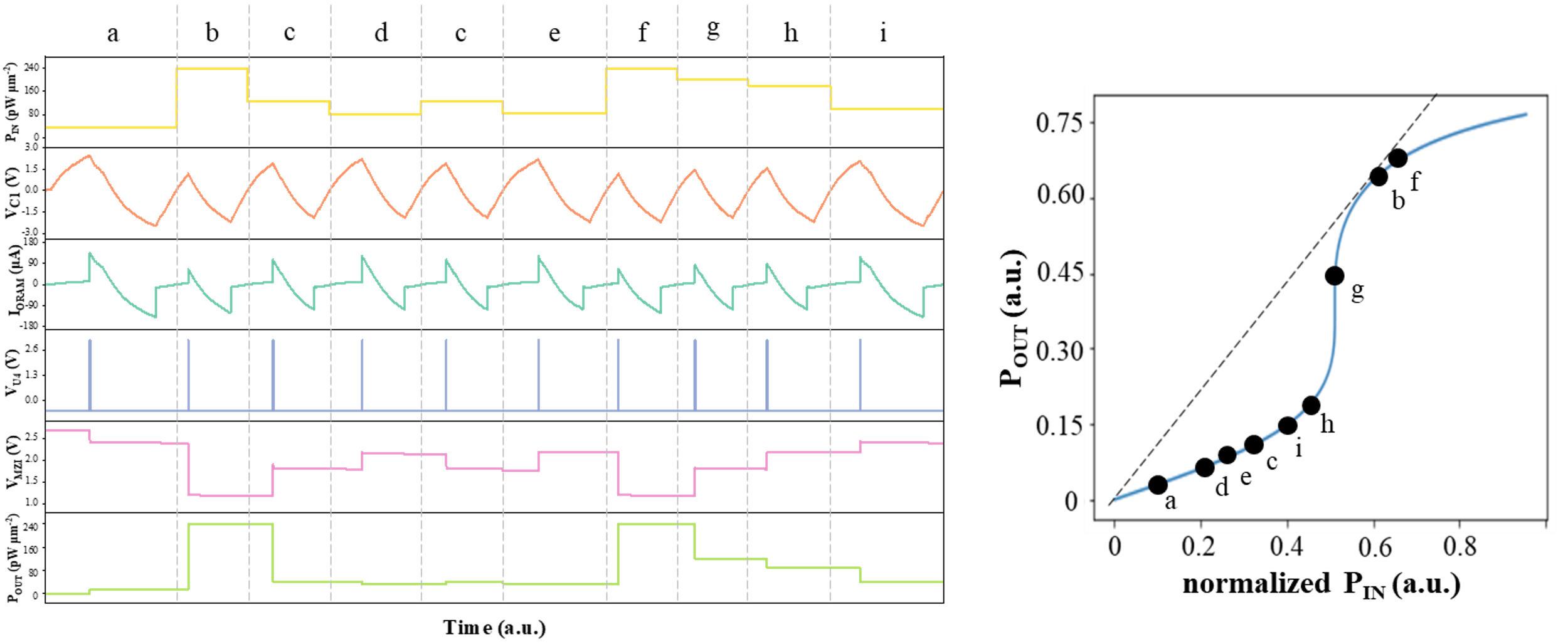


**Fig. S7** Time-series diagram of data obtained from marked nodes in Fig. 4(b) and corresponding points on sigmoid nonlinear activation function diagram.

**References**

1. Sunny, F.P., Taheri, E., Nikdast, M. & Pasricha, S. A survey on silicon photonics for deep learning. *ACM J. Emerg. Technol. Comput. Syst.* **17**, 1-57 (2021).

2. Talib, M.A., Majzoub, S., Nasir, Q. & Jamal, D. A systematic literature review on hardware implementation of artificial intelligence algorithms. *J. Supercomput.* **77**, 1897-1938 (2021).
